# The *in vitro* remineralizing effect of CPP-ACP and CPP-ACPF after 6 and 12 weeks on initial caries lesion

**DOI:** 10.1590/1678-7757-2018-0589

**Published:** 2019-05-20

**Authors:** Laurent A. M. Thierens, Sophie Moerman, Charlotte van Elst, Chris Vercruysse, Petra Maes, Liesbeth Temmerman, Noëmi M. C. de Roo, Ronald M. H. Verbeeck, Guy A. M. de Pauw

**Affiliations:** 1 Ghent University, Department of Orthodontics, Oral Health Sciences, Ghent, Belgium.; 2 Ghent University, Department of Basic Medical Sciences, Biomaterials Group, Ghent, Belgium.; 3 Vrije Universiteit Brussel (VUB), Faculty of Medicine and Pharmacy, Oral Health Research Group (ORHE), Brussels, Belgium.

**Keywords:** Tooth remineralization, Dental white spots, In vitro

## Abstract

**Objective:**

The aim of this *in vitro* study was to determine the effects of remineralization promoting agents containing casein phosphopeptide-stabilized amorphous calcium phosphate (CPP-ACP), or CPP-ACP in combination with fluoride (CPP-ACPF) on artificial white spot lesions (WSLs) after 6 and 12 weeks.

**Methodology:**

White spot lesions were created on 123 sectioned premolars (246 specimens) with a demineralization solution during a 96 hours pH-cycling regime. Two experimental groups were created: a CPP-ACP group (Tooth Mousse™), and a CPP-ACPF group (Mi Paste Plus™). Additionally, two control groups were created, one using only a conventional toothpaste (1450 ppm fluoride) and another one without any working agents. All teeth were also daily brushed with the conventional toothpaste except the second control group. Tooth Mousse™ and Mi Paste Plus™ were applied for 180 seconds every day. The volume of demineralization was measured with transverse microradiography. Six lesion characteristics regarding the lesion depth and mineral content of WSLs were also determined.

**Results:**

The application of CPP-ACP and CPP-ACPF had a significant regenerative effect on the WSLs. Compared to Control group 1 and 2 the volume of demineralization after 6 weeks decreased significantly for CPP-ACP (respectively *p*<0.001 and *p*<0.001) and CPP-ACPF (respectively *p*=0.001 and *p*=0.003). The same trend was observed after 12 weeks. For the CPP-ACPF group, WSL dimensions decreased significantly between 6 and 12 weeks follow-up (*p*=0.012). The lesion depth reduced significantly after application of CPP-ACP and CPP-ACPF but increased significantly in the Control groups. Mineral content increased for CPP-ACP and CPP-ACPF after an application period of 12 weeks, but this was only significant for CPP-ACP.

**Conclusions:**

Long-term use of CPP-ACP and CPP-ACPF in combination with a conventional tooth paste shows beneficial effects in the recovery of *in vitro* subsurface caries lesions.

## Introduction

The white spot lesion (WSL) is the first clinical sign of dental caries.^1^ It is defined as a subsurface enamel porosity caused by carious demineralization and is clinically presented as a milky white opacity when located on smooth surfaces.^2^ The outermost layer of enamel, covering the lesion, remains relatively intact and appears radiopaque in contact radiographs.^3^ In general, orthodontic patients have significantly more white spot lesions than non-orthodontic patients.^2^ ^–^ ^5^ The prevalence of WSLs prior to an orthodontic treatment ranges from 15% to 40%, whereas the incidence of WSLs occurring during orthodontic treatment ranges between 30% and 70%.^6^ ^,^ ^7^ WSL assessment by means of quantitative light-induced fluorescence (QLF) even reported a prevalence of 96%.^8^

An adequate oral hygiene regime and a diet with low carbohydrate intake are important factors reducing the risk for decalcification and WSL formation.^6^ ^,^ ^9^ ^,^ ^10^ There are many possibilities to intervene in the process of demineralization of enamel to arrest or even reverse the progress of the lesion.^1^

The influence of dairy products (milk, milk concentrates, and cheeses) on caries is known since the 1980's when a topical anticariogenic effect of cheese was demonstrated. The protective effect was attributed to a direct chemical effect of the phosphoprotein casein and calcium phosphate contents.^11^ Casein phosphopeptide can bind calcium and phosphate ions thus forming nanoclusters with amorphous calcium phosphate. These casein phosphopeptide – stabilized amorphous calcium phosphate nanoclusters (CPP-ACP) can keep high-concentration gradients of calcium and phosphate ions and ion pairs within the subsurface lesion. The increase in ion concentration in the lesion fluid results in the formation of hydroxyapatite or fluorapatite via crystal growth, thereby depressing enamel demineralization and enhancing remineralization.^11^ ^–^ ^14^ When adequate levels of calcium and phosphate ions are combined with fluoride ions (CPP-ACPF), it has been shown that this combination can result in substantial remineralization of enamel lesions. Fluoride combined with CPP-ACP is demonstrated to be incorporated into the body of the white spot lesion and is not localized at the outermost surface layer of enamel. The diffusion of fluoride ions together with calcium and phosphate ions deep into the lesion enables substantial crystal growth (remineralization) throughout the body of the lesion.^13^ ^,^ ^15^ ^,^ ^16^

However, several *in vitro* studies detected only limited effects or were even unable to detect any significant clinical effects of CPP-ACP(F). Such discrepancy could be explained by the fact that sufficient time is needed for an effective diffusion of CPP-ACP(F) into the body of a subsurface lesion and a subsequent remineralization. Consequently, the study duration must be long enough so that CPP-ACP(F) can act as a saliva biomimetic. Therefore, the objectives of this study were to determine the remineralizing effect of CPP-ACP and CPP-ACPF compared to a conventional toothpaste (1450 ppm fluoride) and a negative control group, and to evaluate the characteristics of white spot lesions in each group after 6 and 12 weeks.

## Methodology

### Preparation of enamel blocks

One hundred and twenty-three extracted premolars were collected, washed in tap-water, and stored in 0.1% thymol solution at 4°C. Only premolars with a healthy enamel surface were selected for this study. Teeth with visual caries, fluorosis or other hypermineralisation defects, pitting, cracks, hypoplastic areas and enamel irregularities were excluded. Approval was granted by the Ghent University Hospital Ethics Committee (B 670 2010 10019) and informed consents were obtained.

The outer enamel surface was polished using Sof-Lex discs (3M, St Paul, Minnesota, USA) attached to a slow-speed contra-angle hand piece to remove a potentially fluoride-rich layer and to avoid individual enamel differences.^17^ The premolars were sectioned into two parts: a buccal side and a palatal side. In order to divide the tooth, a water-cooled diamond saw was used. To avoid interference of general tissue demineralization of the crown, the entire part was covered by acid-resistant nail varnish except on the area of interest.

### Artificial white spot lesions

White spot lesions were created with a demineralization solution of ten Cate and Duijsters (2.2 mM CaCl_2·_2H_2_0 + 1.1 mM NaH_2_PO_4_ + 50 mM CH_3_COOH; pH was adjusted to pH 4.4 using 1 M KOH) during 96 hours to have a lesion depth of 150–200 μm on every half premolar.^18^ This method produces a subsurface enamel demineralization without surface erosion.

### pH-cycling regime

The dynamic model concept of ten Cate and Duijsters with alternating periods of demineralization and remineralization was used in this research project.^18^ The pH-cycling protocol simulates an *in vivo* high caries risk condition and measures simultaneously the net result of the inhibition of demineralization and the enhancement of remineralization. The solutions approximate the mineral ion composition and super saturation of saliva. The remineralization and demineralization solutions in the pH-cycling regime were renewed every day.

Demineralization was performed during 6 hours per day at 37°C (20 mL/sample). An acid buffer containing demineralized H_2_O with 2 mM Ca(Ca(NO_3_)_2_), 2 mM PO_4_ (KH_2_PO_4_) and 75 mM acetate was used. pH was adjusted to 4.3 using 1 M KOH.

Remineralization was performed for 17 hours per day at 37°C (20 mL/sample) using calcium and phosphate concentrations at a known degree of saturation (1.5 mM Ca and 0.9 mM PO_4_) to mimic the remineralizing properties of saliva. This solution contained demineralized H_2_O with 130-150 mM KCl (to provide background ionic strength) 100 mM TRIS, 1.5 mM Ca(NO_3_)_2·_4H_2_0, 0.2 mM KH_2_PO_4_, and 140 mM KCl. pH was adjusted to 7.0 using 1 M HCl.

Prior to the actual start of the experiment, all 246 sections underwent the pH-cycling regime for 3 days without the fluoride toothpaste application to allow baseline values of mineral uptake and loss to be determined. After these 3 days brushing of the teeth was started.

### Study and control groups

The premolar parts were divided in four groups. The prophylactic protocol and the application of remineralisation promoting agents as described by the manufacturer are shown in Table 1. Each of the four groups was divided into 2 subgroups with 6 or 12 weeks follow-up, respectively.

**Table 1:** Prophylactic protocol and descriptive statistics of the study sample

Study group	Prophylactic protocol	6 weeks follow-up		12 weeks follow-up	
		Sections (n)	Sections included in quantitative analysis (n)	Sections (n)	Sections included in quantitative analysis (n)
CPP-ACP	Brushing with conventional toothpaste (Oral B (1100 ppm Stannous Fluoride and 350 ppm Sodium Fluoride)) (every 24 hours) and application of Tooth Mousse™ (every 24 hours for 180 seconds, after brushing)	34	19	36	8
CPP-ACPF	Brushing with conventional toothpaste (Oral B (1100 ppm Stannous Fluoride and 350 ppm Sodium Fluoride)) (every 24 hours) and application of Mi Paste Plus™ (every 24 hours for 180 seconds, after brushing)	26	11	40	12
Control group 1	Brushing with conventional toothpaste (Oral B (1100 ppm Stannous Fluoride and 350 ppm Sodium Fluoride)) (every 24 hours)	17	12	8	7
Control group 2	No working agents	43	26	42	18
TOTAL		120	68	126	45

Legends: n, number; CPP-ACP, casein phosphopeptide-amorphous calcium phosphate; CPP-ACPF, combination of casein phosphopeptide-amorphous calcium phosphate and fluoride

### Volume quantification of demineralization, mineral content, and caries lesion depth

**Figure 1:**
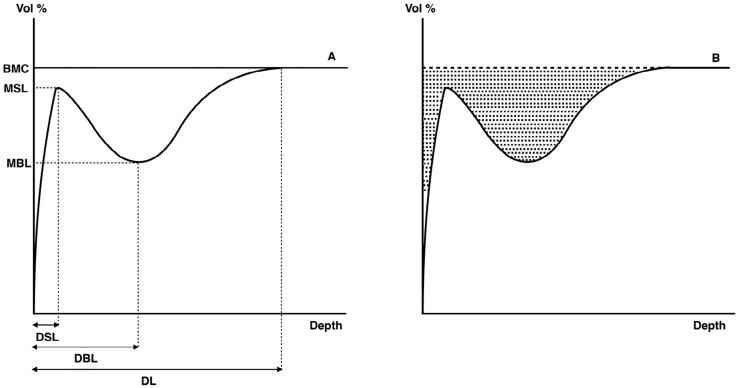
Schematic drawing of white spot lesion using transverse microradiography (based on Theuns, et al.^20^). A) Lesion characteristics: BMC, basic mineral content; MSL, Maximum mineral content of the surface layer; DSL, depth at the point where maximum mineral content is reached; MBL, minimum mineral content of the body lesion; DBL, depth at the point where minimum mineral content is reached; and DL, the depth of the lesion; B) Dissolved minerals are presented as the shaded area

Transverse microradiography (TMR) was used for the quantification of mineral content and caries lesion characteristics.^19^ At least three different enamel sections were cut across the lesion window perpendicular to the enamel surface. These sections were X-rayed with an aluminium stepwedge (PW 1830) at 20 kV and 15 mA for 6 minutes (Philips, Eindhoven, The Netherlands). Only white spot lesions exhibiting an intact surface zone were included. The digitalized images were analyzed with the Sigma Scan Pro software, which calculated the relative grayscale value across a lesion relative to data gathered from sound enamel. The relative mineral content for the corresponding depth was then deduced from the obtained grayscale value. The characteristics of each lesion were determined using the parameters as described by Theuns, et al.^20^ (1986). In Figure 1, these parameters are visually presented in a chart with the mineral content (volume fraction of the mineral) plotted against the depth of the lesion. The average of three measurements for each white spot lesion was used for each parameter.

### Statistics

#### Volume of demineralization

An univariate 2-way ANOVA was used to compare the mean differences of the volume of demineralization between the experimental and control groups, and between 6 and 12 weeks follow-up. In order to avoid a type-1 error, the significance value was adjusted by the Bonferroni correction. *Post-hoc* testing was conducted by using an independent samples t-test to evaluate the single effect of time and treatment. Normality and homogeneity of variances were checked.

#### Lesion characteristics

An independent, non-parametric sample test (Kruskal-Wallis test) was used to detect significant differences in MSL, DSL, MBL, DBL, DL, and BMC 1) after 6 and after 12 weeks, and 2) within each study group between 6 and 12 weeks of follow-up. *Post-hoc* testing was done with the Kruskal-Wallis test with pairwise comparisons of the significant characteristics.

## Results

### Descriptive statistics

By sectioning the premolars, some of the samples became useless due to fracturing of the outer surface of the white spot lesion. The final study sample for 6 and 12 weeks follow-up is shown in Table 1.

### Comparative statistics

#### Volume of demineralization

The assumptions for homogeneity (p>0.05) and normality after log-transformation (p>0.05) are met for every combination.

Table 2 shows the means and standard deviations of the logarithmic volume of demineralization of the four study groups. Significant differences between the groups are indicated with the corresponding *p*-value. A highly significant lower volume of demineralization is observed for the two experimental groups compared to the control groups. This is observed for both 6 and 12 weeks follow-up.

**Table 2:** Means and standard deviations of the logarithmic demineralization volume and of the lesion characteristics after 6 and 12 weeks follow-up. Significant differences are indicated with the corresponding significance level

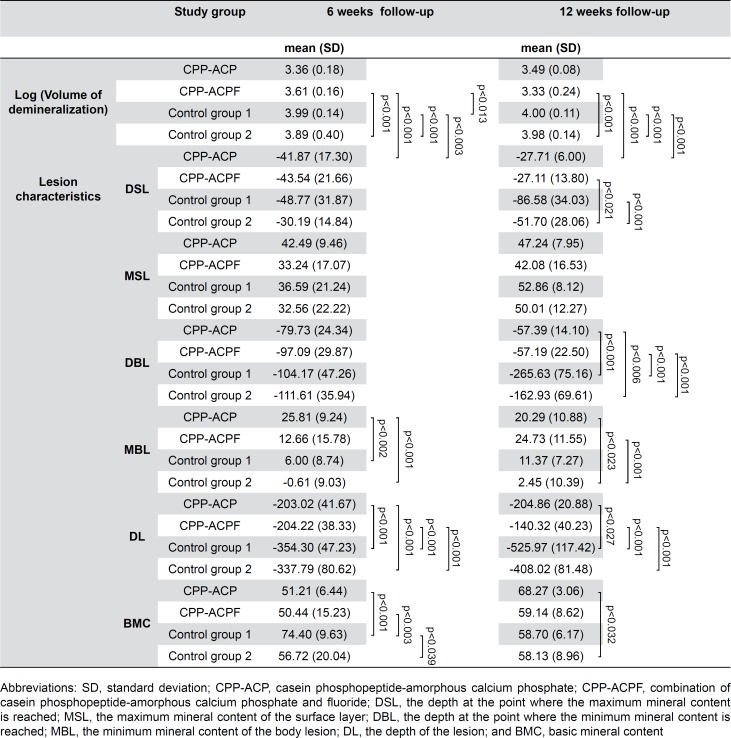

**Figure 2:**
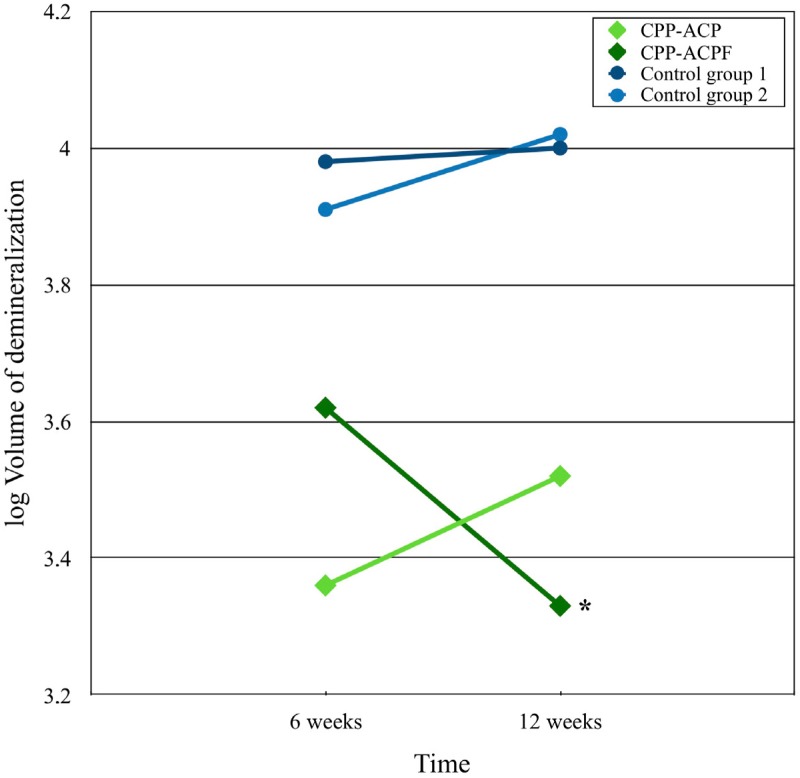
The effect of time and treatment on the logarithmic volume of demineralization of the two experimental and two control groups. The significant reduction of the logarithmic demineralization volume of the CPP-ACPF group from 6 to 12 weeks (*p*=0.012) is indicated with an asterisk (*)

*Post-hoc* testing shows the single effects of the type of treatment and the single effect of time (6 vs. 12 weeks follow-up). The difference between the experimental groups (CPP-ACP vs. CPP-ACPF) was significant after 6 weeks (*p=*0.013) and not significant after 12 weeks (*p=*0.126). There was no significant difference between control group 1 and control group 2, both after 6 and 12 weeks (*p>*0.05). The effect of time was only significant for CPP-ACPF, showing more remineralization after 12 weeks (*p=*0.012), which is clearly illustrated in Figure 2.

#### Lesion characteristics

Table 2 shows the means and standard deviations of the lesion characteristics of the four study groups. Significant differences between groups are indicated with the corresponding *p*-value. After 6 weeks, significant differences occur for MBL (*p<*0.001), DL (*p<*0.001), and BMC (*p<*0.001). *Post-hoc* testing of these significant lesion characteristics shows significant differences, primarily between the experimental groups CPP-ACP and/or CPP-ACPF at one side, and control groups 1 and/or 2 at the other side. A significant difference is also determined between control groups 1 and 2 for BMC (*p=*0.039).

After 12 weeks, significant differences occur for DSL (*p=*0.001), DBL (*p<*0.001), MBL (*p<*0.001), DL (*p<*0.001), and BMC (*p=*0.039). *Post-hoc* testing of these significant lesion characteristics shows similar results compared to 6 weeks follow-up. Significant differences are detected between the experimental groups CPP-ACP and/or CPP-ACPF at one side, and control groups 1 and/or 2 at the other side.

The differences within each study group between 6 and 12 weeks follow-up for the lesion characteristics are shown in Table 3. CPP-ACP significantly reduces the characteristics DSL (*p=*0.029), DBL (*p=*0.034), and increases the BMC (*p<*0.001). CPP-ACPF shows an increase in MBL (*p=*0.042) and significantly reduces the characteristics DBL (*p=*0.002) and DL (*p=*0.010). On the other hand, Control group 1 shows a significant increase in DSL (*p=*0.043), DBL (*p=*0.001), DL (*p=*0.002), and a reduction in BMC (*p=*0.004). Control group 2 also shows a significant increase in DSL (*p=*0.008), MSL (*p=*0.001), DBL (*p=*0.007), and DL (*p=*0.008).

**Table 3:** *p*-values of pairwise comparisons between the 6 and 12 weeks of follow-up for the four study groups regarding lesion characteristics, based on the estimated marginal means. The significance level was adjusted by Bonferroni correction. The bold values represent statistically significant values (*p*<0.05)

	CPP-ACP	CPP-ACPF	Control group 1	Control group 2
DSL	**0.029**	0.056	**0.043**	**0.008**
MSL	0.203	0.196	0.128	**0.001**
DBL	**0.034**	**0.002**	**0.001**	**0.007**
MBL	0.167	**0.042**	0.272	0.206
DL	0.853	**0.010**	**0.002**	**0.008**
BMC	**<0.001**	0.268	**0.040**	0.633

Legends: CPP-ACP, casein phosphopeptide-amorphous calcium phosphate; CPP-ACPF, combination of casein phosphopeptide-amorphous calcium phosphate and fluoride; DSL, the depth at the point where the maximum mineral content is reached; MSL, maximum mineral content of the surface layer; DBL, the depth at the point where the minimum mineral content is reached; MBL, the minimum mineral content of the body lesion; and DL, the depth of the lesion; and BMC, basic mineral content

## Discussion

Long-term application of CPP-ACP(F) combined with a conventional tooth paste reduced the amount of demineralization of artificial white spot lesions (Table 2). The amount of demineralization was comparable after 6 and 12 weeks for both control groups and for the CPP-ACP application. However, the application of CPP-ACPF resulted in a pronounced decrease in demineralized volume between 6 and 12 weeks (Figure 2). Apparently, an additional remineralization occurred mediated by the presence of fluoride ions. The reduction of the demineralization volume after application of CPP-ACP and CPP-ACPF was mainly due to an increase in the mineral content in the body of the lesion (MBL, Table 2) and a concomitant decrease in lesion depth especially after 12 weeks when compared to the control groups (DL, Table 2). On the other hand, the mineral content of the enamel layer covering the lesion remained unchanged (MSL, Table 2). These results clearly indicate that the long-term application of CPP-ACP(F) needs time to take effect, suggesting that diffusion phenomena as well as the mineral deposition in the body of a lesion play an important role.

The dynamic pH-cycling model was used to simulate the conditions of an oral cavity. This *in vitro* model is well established for this type of research and stands for a high level of scientific control and a relatively low variability^21^. In this way, highly variable pH cycles due to the diet and different habits of the patient as seen in *in vivo* models can be avoided. However, *in vitro* studies such as the current study also have their limitations due to the inability to: simulate complex intraoral conditions; mimic solid surface area/solution ratios or the saliva/plaque fluid composition encountered *in vivo*; simulate time periods of de- and remineralization, which are much faster than those expected to occur in *in vivo* conditions; and to adequately simulate topical use and clearance of products from the oral cavity.^21^ The artificial subsurface lesions were created during a 4-day process^18^, but several other processes with a duration ranging from 5 h,^22^ 4 days,^23^ 5 days^23^ ^,^ ^24^ up to 7 days^25^ have been described.

The study was inherently limited by the dropout rate in the study sample. Sectioning of the premolars resulted in fracturing of the outer surface of the white spot lesion and the subsequent inability to quantify lesion depth and mineral content with Transverse Microradiography (TMR). Micro-Computed Tomography (micro-CT) is a non-destructive alternative to quantify mineral density in enamel or dentin.^26^ Research comparing TMR and micro-CT concluded that changes in mineral content and lesion depth are detected with a similar magnitude.^19^

The duration of similar *in vitro* studies varies, going from 10 days^11^ ^,^ ^13^ ^,^ ^27^ up to 21 days^23^ ^,^ ^28^. This is in contrast with the 6 and 12 weeks used in the current study. *In vitro* studies with a duration of 12 weeks are labor intensive, but this approach also yielded new insights. Both volume and mineral content showed significant improvement going from 6 to 12 weeks of follow-up.

The remineralizing potential of CPP-ACP and CPP-ACPF as observed in the current study has also been observed in other *in vitro* studies,^11^ ^,^ ^25^ ^,^ ^29^ *in situ* studies,^15^ ^,^ ^30^ and *in vivo* studies.^31^ ^,^ ^32^ However, Pulido and coworkers^26^ (2008) observed no significant differences between the effects of CPP-ACPF and artificial saliva on the inhibition of a lesion progression in an *in vitro* setting. The absence of any significant effects could be attributed to the short application period of 2 minutes, instead of the 3 minutes prescribed by the manufacturer.^24^ The application of CPP-ACP(F) requires sufficient time to enable effective diffusion of nanoclusters through the surface layer of the white spot lesion. More recent studies, both *in vitro* and *in vivo*, were also unable to confirm the potential of CPP-ACP and CPP-ACPF in conjunction with a conventional toothpaste.^28^ ^,^ ^33^ ^,^ ^34^ On the other hand, the prescribed application time of 3 minutes of CPP-ACP(F) must not be extended to produce an additional effect. *In vitro* research by Vieira and coworkers^37^ (2017) did not show superior mineral gain or lesion depth reduction compared to a placebo group after increase in the application time up to 3 and 8 hours.

Beerens and coworkers^38^ (2017) could not detect favorable effects of CPP-ACPF in a randomized clinical trial of 12 months with orthodontic patients after bracket removal. The clearance time from the oral cavity might be a contributing factor for the reduced *in vivo* effect. Fluoride treatments show a rapid clearance in the oral cavity because the major fraction of fluoride is lost when spitting out the excessive saliva. A similar reduction might be seen after the application of CPP-ACP(F) pastes, because a similar procedure is recommended by the manufacturer. In this respect, the remineralizing agents are unable to show their full potential.^35^ In order to deal with the clearing effect of saliva, lozenges or chewing gum containing CPP-ACP(F) are an alternative for creams. *In situ* randomized research showed that sugar free gum containing CPP-ACP promoted greater remineralization levels.^36^

No significant differences between CPP-ACP and CPP-ACPF could be detected for the volume of demineralization, mineral content, or lesion depth. The adjustment of the pH to 7.0 during remineralization might explain this. Similar levels of remineralization produced by CPP-ACP and CPP-ACPF solutions from pH 7.0 to 6.0 were also observed by Cochrane and coworkers^40^ (2012). However, CPP-ACPF solutions outperformed CPP-ACP solutions when pH dropped to 5.5, 5.0, and 4.5. This effect was likely attributed to the presence of fluoride.^13^

## Conclusion

CPP-ACP and CPP-ACPF in combination with a conventional tooth paste (1450 ppm F) reduce the extent and increase the mineral content of *in vitro* subsurface caries lesions. An application period of up to 12 weeks of CPP-ACPF showed superior results compared to a shorter application period (6 weeks). The latter emphasizes the importance of prolonged use in order to reduce the white spot lesion.
